# l-Ornithine affects peripheral clock gene expression in mice

**DOI:** 10.1038/srep34665

**Published:** 2016-10-05

**Authors:** Takafumi Fukuda, Atsushi Haraguchi, Mari Kuwahara, Kaai Nakamura, Yutaro Hamaguchi, Yuko Ikeda, Yuko Ishida, Guanying Wang, Chise Shirakawa, Yoko Tanihata, Kazuaki Ohara, Shigenobu Shibata

**Affiliations:** 1 Research Laboratories for Health Science & Food Technologies, Kirin Company, Ltd., Yokohama, Kanagawa, Japan; 2 Laboratory of Physiology and Pharmacology, School of Advanced Science and Engineering, Waseda University, Tokyo, Japan

## Abstract

The peripheral circadian clock is entrained by factors in the external environment such as scheduled feeding, exercise, and mental and physical stresses. In addition, recent studies in mice demonstrated that some food components have the potential to control the peripheral circadian clock during scheduled feeding, although information about these components remains limited. l-Ornithine is a type of non-protein amino acid that is present in foods and has been reported to have various physiological functions. In human trials, for example, l-ornithine intake improved a subjective index of sleep quality. Here we demonstrate, using an *in vivo* monitoring system, that repeated oral administration of l-ornithine at an early inactive period in mice induced a phase advance in the rhythm of PER2 expression. By contrast, l-ornithine administration to mouse embryonic fibroblasts did not affect the expression of PER2, indicating that l-ornithine indirectly alters the phase of PER2. l-Ornithine also increased plasma levels of insulin, glucose and glucagon-like peptide-1 alongside m*Per2* expression, suggesting that it exerts its effects probably via insulin secretion. Collectively, these findings demonstrate that l-ornithine affects peripheral clock gene expression and may expand the possibilities of L-ornithine as a health food.

The circadian clock system plays an important role in maintaining physiological functions including the sleep-wake cycle, body temperature, metabolism and various organ functions^1^. Intensive studies have shown that this ~24-h cycle is generated by a transcriptional feedback loop of clock genes. Representatively, a heterodimer of the proteins CLOCK and BMAL1 promotes transcription of the genes *Per1*, *Per2*, *Cry1* and *Cry2*, which in turn suppress *Clock* and *Bmal1* expression^2^. Clock genes bind specific DNA elements, namely the E- and E’-boxes, which regulate the expression of various genes. A previous report indicated that the clock genes feedback loop might affect up to 10% of mammalian mRNA expression in the liver^3^.

In the mammal circadian system, the suprachiasmatic nuclei (SCN) in the brain are thought to be the master pacemaker of circadian rhythm. In addition, almost all peripheral tissues possess circadian clocks^4^. On the one hand, the central clock in SCN is synchronized by mainly light-dark stimuli, and regulates the peripheral clocks through behavioural, endocrine and neural pathways^5^,^6^. On the other hand, recent research has revealed that other physiological stimuli, including pharmacological agents, nutrients, and mental and physical stresses, affect the peripheral clocks^7^,^8^. In particular, the effects of food components on circadian clock have been recently receiving attention. For example, it has been reported that caffeine lengthens the period and causes a phase shift of peripheral clocks in mice^9^. Another report has suggested that food containing fish oil promotes m*Per2* expression and also causes a phase shift in mice^10^. In addition to these reports, it was recently reported that Nobiletin, a natural polymethoxylated flavone, enhanced the amplitude of circadian oscillation by binding to specific elements of clock genes^11^.

l-Ornithine is a naturally occurring type of non-protein amino acid found in foods such as corbicula, cheese and flounder. l-Ornithine is generated in the urea cycle in the liver and kidney. In addition, various physiological functions of l-ornithine have been reported. For instance, l-ornithine intake was found to increase non-rapid eye movement sleep time in mice^12^. Furthermore, trials in healthy human revealed that l-ornithine intake improves a subjective index for sleep quality and attenuates the serum cortisol/DHEA-S molar ratio, which is a measure of stress^13^,^14^,^15^. Physiological effects of l-ornithine on the circadian rhythm have been also reported. It has been demonstrated that L-ornithine affects plasma growth hormone, melatonin, and corticosterone levels in mice, and its effects were found to depend on administration time^16^. However, data on the molecular mechanisms underlying these physiological effects of l-ornithine remain limited. In this study, we examined whether oral administration of l-ornithine affects peripheral clock gene expression in mice in order to determine the mechanism underlying the previously reported effects of l-ornithine.

## Results

### Administration of l-ornithine advanced the PER2 expression phase in mice

**Figure 1:**
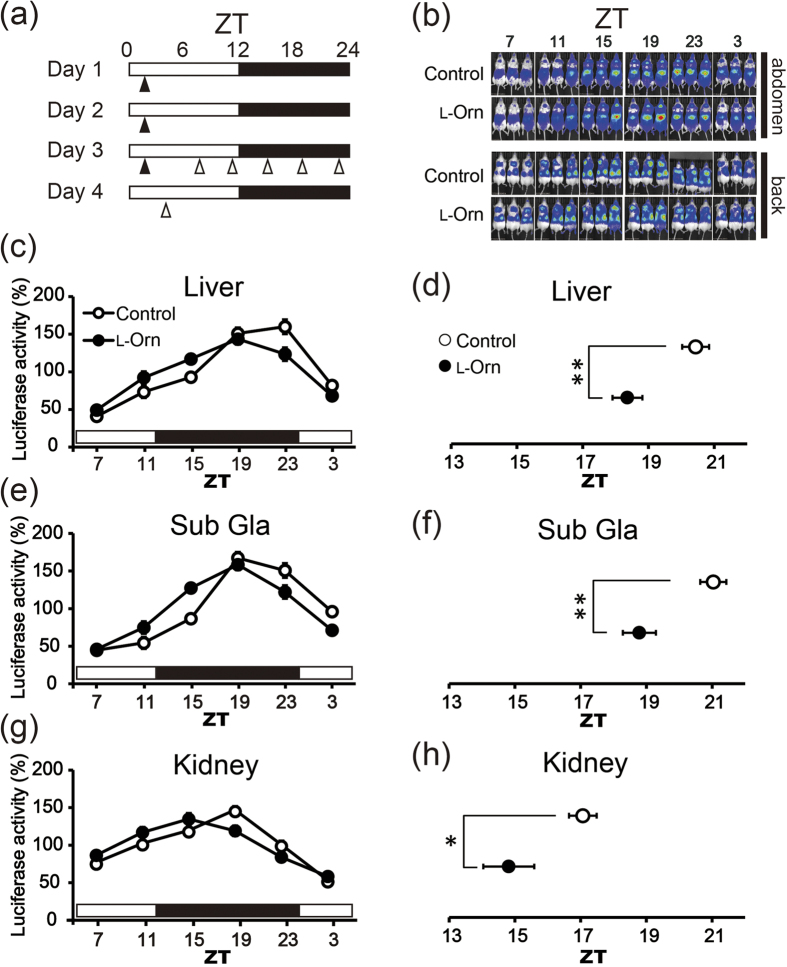
L-ornithine administration at Zeitgerber time 1 (ZT 1) causes a phase advance of PER2 rhythm, as evaluated by *in vivo* monitoring. (**a**) Experimental schedule. Open and filled bars indicate light and dark periods, respectively. At ZT 1, l-ornithine (1,000 mg/kg) or vehicle was administered to mice for three consecutive days by oral injection (filled arrowhead). Luciferase activity was measured six times every four hours (open arrowheads). (**b**) Representative photographs from each time point at 4-h intervals (three PER2::LUC mice were assessed simultaneously per time point). Upper panels show the ventral position with images of the liver and sub gla; lower panels show the prone position with images of the kidney. (**c**,**e**,**g**) Oscillatory PER2::LUC bioluminescence in the liver, sub gla and kidney of mice treated with l-ornithine (filled circles) or vehicle (open circles) (mean ± SEM: n = 15 mice, l-ornithine; n = 12 mice, vehicle). (**d**,**f**,**h**) Average peak phase of PER2::LUC bioluminescence in the liver, sub gla and kidney calculated by the cosinor procedure program. *P < 0.05, **P < 0.01 versus control (Mann-Whitney *U* test).

To investigate whether l-ornithine administration affects the peripheral circadian clock, we evaluated the circadian phase of PER2::LUC expression in the liver, sub gla and kidney of mice by utilizing an *in vivo* monitoring system. Previous reports have suggested that the effect of scheduled feeding on circadian rhythm is dependent on feeding timing in mice^17^,^18^. For example, Hirao *et al*. reported that scheduled feeding at ZT 0–3 affects the phase of circadian clocks more strongly than later feeding^19^. In accordance with this report, we determined the effect of l-ornithine administration at ZT 1. Figure 1a shows the time schedule of the experiment. Throughout this experiment, mice could access food and water *ad libitum*. l-Ornithine (1,000 mg/kg) was administered to mice for three consecutive days by oral injection at ZT 1; on the last day, bioluminescence intensity due to luciferase, which reflects the expression level of PER2::LUC, was measured six times at 4-h intervals with the animal in the supine and prone position (Fig. 1b). Luciferase activities were plotted against ZT (Fig. 1c,e,g), and peak phases were calculated by the single cosinor procedure program (Fig. 1d,f,h). As compared with vehicle controls, the mice treated with l-ornithine-treated at ZT 1 showed a significant phase advance of bioluminescence emitted by PER2::LUC rhythms in the liver, sub gla, and kidney (Fig. 1c–h).

### l-Ornithine effects on peripheral clock genes detected by time series RT-PCR

**Figure 2:**
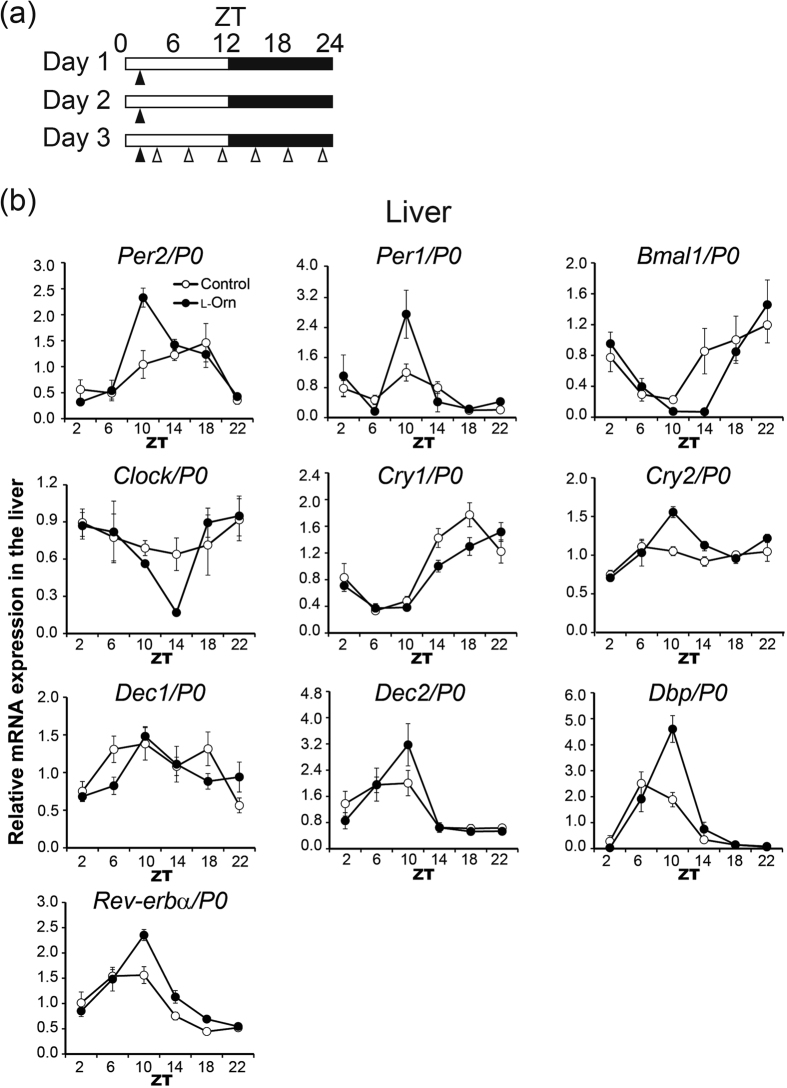
Rhythm of clock gene mRNA expression in the liver detected by time series RT-PCR. (**a**) Experimental schedule. Open and filled bars indicate light and dark periods, respectively. At ZT 1, l-ornithine (1,000 mg/kg) or vehicle was administered to mice for three consecutive days by oral injection (filled arrowheads). Mice were killed at 4-h intervals throughout the day (open arrowheads). (**b**) mRNA levels of clock genes in the liver determined by RT-PCR plotted against ZT. All values are expressed as means ± SEM (n = 5–9 mice per group).

It was not possible to evaluate the expression rhythm of clock genes other than PER2 by the *in vivo* monitoring method using the PER2::LUC knock-in mice. Therefore, to examine whether l-ornithine affects not only m*Per2* but also other clock genes (i.e., m*Per1*, m*Clock*, m*Bmal1*, m*Cry1*, m*Cry2*, m*Dec1*, m*Dec2*, m*Dbp and* m*Rev-erbα*), we carried out time series RT-PCR. As in the *in vivo* monitoring experiment, l-ornithine (1,000 mg/kg) was administered to mice for three consecutive days by oral injection at ZT 1, and the liver, sub gla and kidney were collected at different time points (ZT 2, ZT 6, ZT 10, ZT 14, ZT 18 and ZT 22; n = 5–9 mice per time point) (Fig. 2a). The oscillation of clock genes were determined by RT-PCR and the phase shift was evaluated by determining the acrophase by fitting to a cosinor curve.

Overall, the phase of the expression of m*Per2* in the liver and kidney was advanced, consistent with the *in vivo* monitoring data (Fig. 2b and Supplementary Fig. S2, Table 1 and Supplementary Table S2). On the other hand, the acrophase of m*Per2* in the sub gla was the same in the control and l-ornithine groups (Supplementary Fig. S1 and Supplementary Table S2). However, the peak position of the expression of m*Per2* in the sub gla was advanced in the l-ornithine-treated group as compared with the control group, weakly suggesting that m*Per2* rhythm in the l-ornithine-treated group tended to advance. The phase of the expression of clock genes other than m*Per2* was almost unaffected by l-ornithine administration.

### l-Ornithine effects on clock genes in MEFs detected by real-time PER2::LUC assay

**Figure 3:**
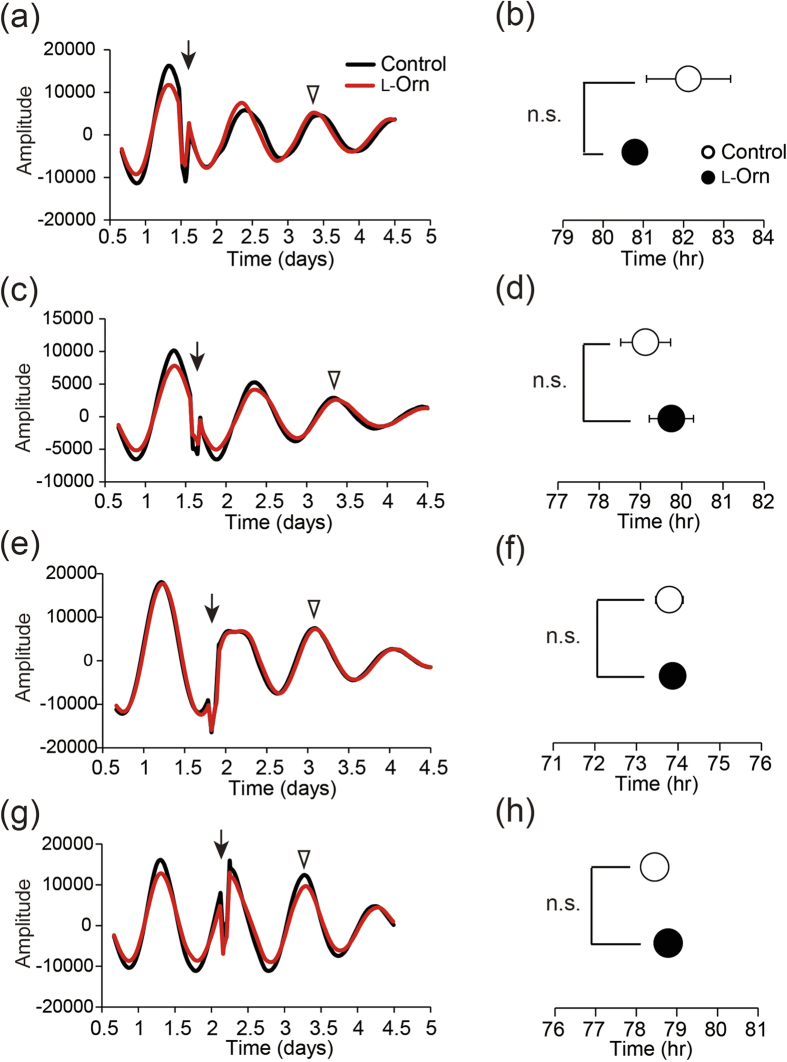
Effect of L-ornithine on PER2::LUC oscillation in MEFs. (**a**,**c**,**e**,**g**) Representative data for MEFs treated with water or 1 mM l-ornithine. Filled arrows indicate the time point of l-ornithine application; open arrowheads indicate the peak phase. (**b**,**d**,**f**,**h**) Summary of the peak phase of m*Per2* expression. All values are expressed as means ± SEM (n = 4 mice per group).

Next, we examined whether l-ornithine administration affects clock genes in the peripheral organs of mice directly or indirectly. To address this question, we evaluated whether l-ornithine alters PER2 expression pattern directly by using a real-time PER2::LUC assay in MEFs derived from PER2::LUC knock-in mice. We considered that if l-ornithine is absorbed by the cells to affect PER2 expression directly, we would observe the same effects as in the *in vivo* experiment. Therefore, l-ornithine or vehicle was added to the MEFs at four different time points. As shown in Fig. 3, there was no significant difference in peak phase between the l-ornithine- and vehicle-treated groups at any time point of l-ornithine treatment. This result indicates that l-ornithine alters the phase of PER2 oscillation indirectly by, for example, hormone secretion.

### Administration of l-ornithine at ZT 1 induces secretion of insulin

**Figure 4:**
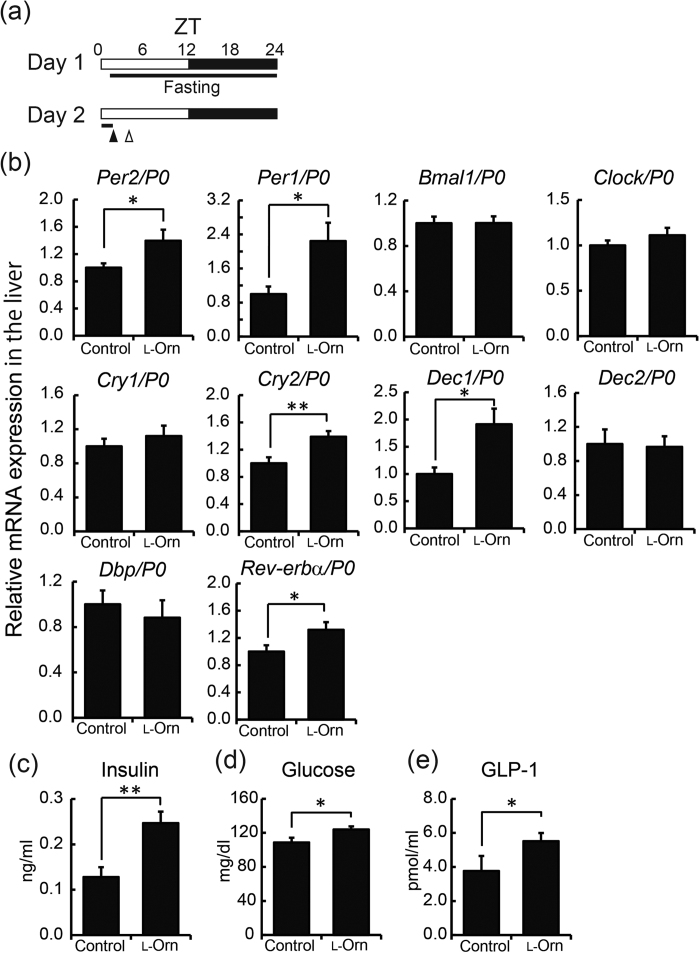
Effects of L-ornithine administration at ZT 1 on secretion of insulin, GLP-1, and glucose in plasma, and clock genes in the liver. (**a**) Experimental schedule. Open and filled bars indicate light and dark periods, respectively. Mice were entrained to the light-dark cycle for at least 14 days. At ZT 1, l-ornithine (1,000 mg/kg) or vehicle was administered to mice after 24 h fasting by oral injection (filled arrowhead); at ZT 2, plasma and tissues were collected (open arrowhead). (**b**) RT-PCR analysis of clock genes in the liver. mRNA expression was normalized to *P0*. (**c**–**e**) Insulin, glucose and total length GLP-1 concentration in plasma (mean ± SEM, n = 10 mice respectively). **P < 0.01, *P < 0.05 versus control (Mann-Whitney *U* test).

Insulin secretion is reported to play an important role in circadian entrainment by food intake, especially in the liver^20^,^21^. Therefore, we focused on clock genes in the liver under the hypothesis that insulin is a “mediator” for the l-ornithine-induced effects on clock genes. First, after 24 h fasting to avoid any dietary influence, mice were given l-ornithine or vehicle at ZT 1, and plasma and the liver samples were collected 1 h later (Fig. 4a). Relative to the control group, l-ornithine significantly increased expression levels of m*Per2*, m*Per1*, m*Cry2*, m*Dec1* and m*Rev-erbα* (Fig. 4b). Furthermore, l-ornithine significantly increased plasma insulin levels (Fig. 4c). We also investigated levels of glucose and glucagon-like peptide-1 (GLP-1) in plasma, because these two factors are known to affect insulin secretion. As shown in Fig. 4d,e, glucose and GLP-1 levels were also increased by l-ornithine. To determine whether the effects on clock genes were specific to l-ornithine, we evaluated the influence on m*Per2* of two other amino acids: l-glutamic acid (1,000 mg/kg), which is a precursor of l-ornithine; and l-arginine (1,000 mg/kg), which is a component of the urea cycle. As shown in Supplementary Figure S3, l-ornithine significantly increased m*Per2* and insulin levels as compared with the control, whereas both l-glutamic acid and l-arginine increased plasma insulin and m*Per2* expression levels slightly but not significantly.

**Figure 5:**
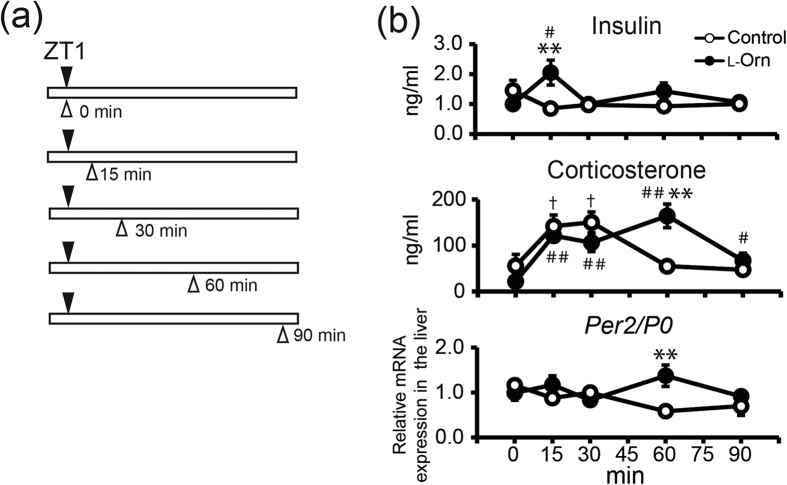
Time course of insulin, corticosterone, and m*Per2* mRNA expression induced by L-ornithine administration in the non-fasted condition. (**a**) Experimental schedule. Mice were entrained to the light-dark cycle for at least 14 days. At ZT 1, l-ornithine (1,000 mg/kg) or vehicle was administered to mice (filled arrowhead), and plasma and tissue samples were collected after 0, 15, 30, 60, and 90 min (open arrowhead). (**b**) Time course of insulin and corticosterone in plasma, and m*Per2* mRNA (normalized to *P0*) in the liver (mean ± SEM, n = 9–10 mice respectively). **P < 0.01, *P < 0.05 versus control (Mann-Whitney *U* test), ^##^*P* < 0.01, ^#^*P* < 0.05 versus 0-min value (l-ornithine-treated group, Steel test), ^†^*P* < 0.05 versus 0-min value (control group, Steel test).

Next, we examined m*Per2* and insulin levels at more time points in the non-fasted condition to clarify in more detail the relationship between m*Per2* and insulin (Fig. 5). In addition, we evaluated corticosterone levels after l-ornithine administration, because corticosterone and dexamethasone are known to cause the entrainment of peripheral clocks^7^,^22^,^23^. l-ornithine (1,000 mg/kg) was administered to mice at ZT 1, and the liver, sub gla and kidney were collected at different time points (administration after 0, 15, 30, 60 and 90 min, 9–10 mice per time point) (Fig. 5a). As shown in Fig. 5b, in the l-ornithine-treated group, the insulin level increased acutely after 15 min of administration and, subsequent to the secretion of insulin, expression of m*Per2* increased after 60 min of administration. Corticosterone levels were increased after 15 and 30 min in both groups as compared with levels at 0 min. At 60 min after administration, the corticosterone level in the l-ornithine-treated group was higher than that in the control group.

## Discussion

We have shown that repeated administration of l-ornithine at ZT 1 induced a phase advance of PER2 expression in PER2::LUC mice by utilizing an *in vivo* monitoring system (Fig. 1). We first hypothesized that l-ornithine might be absorbed intracellularly to directly affect clock genes transcription, protein degradation, and so on; however, application of l-ornithine to MEFs did not affect the phase of PER2 expression *in vitro* (Fig. 3). This result suggested that l-ornithine might affect the expression of clock genes indirectly.

Next, we focused on l-ornithine-induced insulin secretion, because insulin is known to be a powerful entraining hormone^20^,^24^, and a previous study demonstrated that l-ornithine stimulates intestinal l-cells and induces the secretion of GLP-1 via G protein-coupled receptors *in vitro*^25^. We therefore examined whether oral administration of l-ornithine at ZT 1 after fasting could induce the secretion of GLP-1, glucose and insulin in mice (Fig. 4). We demonstrated that l-ornithine administration increased insulin secretion alongside m*Per2* expression. According to previous reports, insulin treatment promotes the expression of m*Per1*, m*Cry2*, m*Dec1* and m*Rev-erbα* in addition to m*Per2*^24^,^26^. As shown in Fig. 4, the expression of these clock genes in the liver, which is highly sensitive to secreted insulin^20^, was also promoted. Furthermore, we examined the time course of expression of m*Per2* in the liver and insulin secretion in detail (Fig. 5). As a result, we demonstrated that the rise in expression of m*Per2* occurred after the rise in insulin level. ZT 1 is near the nadir of PER2 expression rhythm, as shown in Fig. 1; hence, increasing PER2 expression may lead to an advance shift of the PER2 expression phase. On the other hand, it was recently reported that l-ornithine metabolites (putrescinem, spermidine and spermine) affect the circadian period^27^. In all probability, many mechanisms underlie the effects of l-ornithine; therefore, the kidney and sub gla, which have low sensitivity to insulin, were also affected by l-ornithine and/or its metabolites (Fig. 1e–h). For example, we evaluated the corticosterone level in addition to insulin, because corticosterone and dexamethasone injection can entrain the peripheral clock^7^,^22^,^23^. We demonstrated that the corticosterone level had increased in both groups at 15 and 30 min after administration. Corticosterone is often used as a stress marker; therefore, oral administration might have caused stress to the mice and would probably have affected the corticosterone level in both groups. Although corticosterone rose in the control and l-ornithine groups (20–170 ng/ml corticosterone), the level was too small to induce a phase shift of the peripheral clock, because a strong immobile stress (1,500–2,000 ng/ml corticosterone) but not a mild stress (500 ng/ml corticosterone) causes aphase shift of the peripheral clock^7^. Thus, these observations indicated that the contribution of corticosterone was small in the current experiments. Taken together, although the phase advance of PER2 due to insulin secretion might be one of several mechanisms, these results indicate that insulin secretion is involved.

To investigate whether the effect on clock genes after fasting is specific to l-ornithine, we also evaluated two other amino acids, l-glutamic acid and l-arginine. The administration of either amino acid after fasting did not affect clock gene expression (Supplementary Fig. S3b). Interestingly, the expression level of m*Per2* correlated with the concentration of insulin in plasma; this observation also supports the mechanism that we have proposed.

An advance in PER2 expression phase was observed during both *in vivo* monitoring and mRNA analysis (Figs 1 and 2, Supplementary Figs S1 and S2), whereas at the mRNA level, clock genes other than m*Per2* were not affected by administration of l-ornithine (Fig. 2). In our previous studies, the responses of clock gene expression to entrainment stimuli were found to be dependent on peripheral tissues and/or the type of clock gene^7^,^23^. For example, in terms of the rhythm of m*Per2* but not m*Per1*, m*Bmal1* or m*Rev-erbα* mRNA expression, treadmill exercise caused a stronger phase-advance in the kidney, liver and sub gla, as compared with wheel-running, and treadmill and wheel-running exercise produced a similar phase advance in gastrocnemius muscle and lung^23^. Although a single dose of l-ornithine after fasting induced the acute expression of many clock genes in Fig. 4, mouse organs might be more sensitive to secreted insulin after fasting, which would account for this difference. Further experiments are necessary to clarify the mechanism underlying the specific effects of l-ornithine on clock gene expression. In fact, we did not carry out time series RT-PCR analysis or *in vivo* monitoring evaluation under fasting conditions for the following two reasons. First, a previous study showed that decreasing food volume causes a phase advance in peripheral clocks^28^; therefore, it is extremely difficult to evaluate the effect of l-ornithine properly. The second reason is related to animal protection: because these experiments would require a very long fasting period, it was not possible to perform them. If these issues are resolved, we will be able to probe the mechanism more deeply.

In previous trials in humans, l-ornithine intake before bedtime was found to improve sleep quality subjectively^13^,^14^. In this study, we confirmed that l-ornithine administration at ZT 1 (i.e. the early inactive phase of the mice) produced a phase advance of PER2 rhythm. In addition, a previous report suggested that dissociation of the peripheral and central clocks caused a misalignment of daily rhythms in human physiological functions^29^. Although we examined only the peripheral clocks in this study, advancing the phase of the peripheral clock gene *mPer2* might cancel the prolonged sleep-onset latency in humans caused by exposure to evening light^30^. We aim to investigate the relationship between L-ornithine and the central clock in a future study.

## Materials and Methods

### Chemicals

l-Ornithine monohydrochloride was purchased from Kyowa Hakko Bio (Tokyo, Japan). l-Arginine and l-glutamic acid were purchased from Wako (Osaka, Japan).

### Animals

All animal care and experimental procedures were done in accordance with the guidelines of the Committee for Animal Experimentation at Waseda University and Kirin Company. The studies were approved by the Committee for Animal Experimentation at Waseda University and Kirin Company respectively (approval numbers: 2015-A037, Waseda University; YO14-00113, YO15-00023, AN10227-Z00, AN10228-Z01 and AN10229-Z00 Kirin Company). ICR mice purchased from Charles River Laboratories Japan (Kanagawa, Japan) were used for RT-PCR analysis and plasma analysis. Heterogeneous PRE2::LUC knock-in mice^31^,^32^, which were generated by backcrossing ICR mice, were used for *in vivo* monitoring of PER2::LUC bioluminescence. Individually housed male mice aged at least 8 weeks were used in all experiments. Mice were kept in a room maintained on a 12:12 h light-dark cycle at a temperature of 23 ± 1 °C. Zeitgerber time 0 (ZT 0) was defined as lights-on and ZT 12 was defined as lights-off. Mice were provided with a standard mouse chow (EF; Oriental Yeast Company (Tokyo, Japan) for the *in vivo* monitoring experiment, and CE-2 (CLEA Japan, Tokyo, Japan) for the other experiments.

### *In vivo* monitoring of PER2::LUC bioluminescence

At a predetermined time, l-ornithine (1,000 mg/kg) was administered to mice by oral injection (10 mL/kg) for three days, while control mice were injected with water. *In vivo* monitoring of PER2::LUC bioluminescence was performed as previously described^31^ using an *in vivo* imaging system (IVIS) kinetics system (Caliper Life Sciences, Hopkinton, MA, USA). Mice were anaesthetized with isoflurane (Mylan Inc., Tokyo, Japan) and concentrated oxygen (SO-005B; Sanyo Electronic Industries Co. Ltd, Okayama, Japan) in a black box via a gas anaesthesia system (XGI-8; Caliper Life Sciences, Hopkinton, MA, USA). Anaesthetized mice were injected with D-luciferin potassium salt (s.c., 15 mg/kg), and images were acquired with a 1-min exposure time after 6 and 8 min in the prone position for the kidney, and after 10 and 12 min in the supine position for the liver and submandibular gland (sub gla). Each bioluminescence image was merged with the corresponding greyscale image. Images were obtained six times per day (ZT 7, ZT 11, ZT 15, ZT 19, ZT 23 and ZT 3). Mice were returned to their home cages after each imaging session, where they recovered quickly from the anaesthesia. The total time under anaesthesia was approximately 20 min per session. A previous study has shown that LUC activity in the peripheral tissues and animal behaviour are unaffected by four hourly anaesthesia and bioluminescence analysis^31^.

### *In vivo* monitoring data analysis

The bioluminescence emitted from each organ (liver, sub gla and kidney) was calculated by using MetaMorph software (NipponRoper, Molecular Device Japan, Tokyo, Japan). For each individual organ, the region of interest was set to the same shape and size throughout all experiments. The averaged photon per second value of data from the six time points for days 0–1 was designated as 100%, and the bioluminescence rhythm for the entire day was expressed as a percentage of each set of six time points for each individual organ. The peak phase and amplitude of these normalized percentage data were determined by using the single cosinor procedure program (Acro.exe, version 3.5; designed by Refinetti *et al*., 2007).

### RNA isolation and real-time quantitative RT-PCR

Mouse liver mRNA levels were measured by real-time quantitative RT-PCR. The livers were removed after euthanasia, washed in saline, immediately frozen in liquid nitrogen, and stored at −80 °C until RNA isolation. Total RNA was extracted by using TRIzol ® Reagent (Ambion, Waltham, Massachusetts, USA), and purified by using an RNeasy Mini Kit (QIAGEN, Hilden, Germany). To synthesize cDNA, 2.5 μg of purified total RNA was reverse-transcribed by using the SuperScript® VILO™ cDNA Synthesis Kit (Invitrogen, Waltham, MA, USA). Quantification of relative RNA levels was performed with SYBER Green real-time PCR technology (Applied Biosystems, Waltham, MA, USA). Data were normalized to ribosomal protein large *P0* RNA (*P0*). The specific forward and reverse primer pairs are shown in Table 2.

### l-Ornithine treatment and assessment of bioluminescence in PER2::LUC MEFs

The rhythmic expression of PER2 was measured by using a real-time LUC assay in mouse embryonic fibroblasts (MEFs) derived from PER2::LUC knock-in mice^24^. Bioluminescence was monitored once per minute over 10-min intervals with a dish-type luminometer (LumiCycle; Actimetrics, Wilmette, IL, USA). Cell lines were stimulated with 200 nM dexamethasone for 2 h to synchronize PER2 expression rhythms. The medium was replaced with DMEM containing 0.1 mM D-luciferin potassium salt (Promega, Madison, WI, USA), 10% FBS (Bio West, Kansas City, MO, USA) and the specified treatment before measurements were taken. For transient l-ornithine treatment, MEFs were treated with vehicle (water) or l-ornithine (1 mM) for 30 min at 36 h, 37 h, 43 h or 51 h after dexamethasone stimulation. After l-ornithine treatment, the l-ornithine-containing culture medium was replaced with fresh medium, and bioluminescence was measured over 4 days. The phase and amplitude of the second peak after treatment were recorded for analysis.

### Assessment of circadian rhythm in MEFs

Raw data (1-min bins) were smoothed by an adjusting averaging method with 2-h running means, as previously described^33^,^34^. The data were then de-trended by subtracting the 24-h running average from the raw data using R software (R development Core Team; http://www.r-project.org/), created by Mr. Tsuyoshi Yaita and Dr. Shigenobu Shibata (Waseda University, Tokyo, Japan). Details of this assessment have been reported previously^31^. A peak was defined as the point at which bioluminescence was higher as compared with adjacent points, and was confirmed by wave form.

### Measurement of insulin, glucose, corticosterone and GLP-1 levels in mice plasma

Various test substance were administered to mice via oral injection at ZT 1. At ZT 2, plasma was collected from the retro-orbital venous plexus during ether anaesthesia with EDTA-coated blood collection tubes (TERUMO, Tokyo, Japan). To avoid degradation of glucagon-like peptide-1 (GLP-1), the blood collection tubes were pre-added with 5 μl of 30 mM diprotin-A (Bachem, Torrance, CA, USA), which is an inhibitor of dipeptidyl aminopeptidase IV. Plasma samples were immediately frozen in liquid nitrogen and stored at −80 °C until analysis. Insulin, corticosterone and GLP-1 levels were measured by using an ELISA kit (TaKaRa, Kyoto, Japan, Yanaihara, Shizuoka, Japan and IBM, Armonk, NY, U.S. respectively) in accordance with the manufacturer’s instructions.

### Data analysis

Differences between two groups were evaluated by Mann-Whitney *U* test and differences between multiple groups were evaluated by Steel-Dwass test (pairwise comparisons of all groups) or Steel test (pairwise comparison with the control group). A *P* value of <0.05 was considered statistically significant.

## Additional Information

**How to cite this article**: Fukuda, T. *et al*. l-Ornithine affects peripheral clock gene expression in mice. *Sci. Rep.* **6**, 34665; doi: 10.1038/srep34665 (2016).

## Supplementary Material

Supplementary Information

## Figures and Tables

**Table 1:** List of amplitudes, acrophases and *P* values (representing precision of the fit) of the time series RT-PCR data in Fig. 2 calculated by the cosinor procedure program.

Liver				
Clock Gene	Group	Amplitude	Acrophase	*P* value
*Per2*	Control	0.553	14.8	<0.05
	l-Orn	1.004	12.4	<0.05
*Per1*	Control	0.504	9.2	>0.05
	l-Orn	1.296	8.8	>0.05
*Bmal1*	Control	0.485	20.0	<0.001
	l-Orn	0.694	22.0	<0.001
*Clock*	Control	0.139	2.0	<0.02
	l-Orn	0.390	2.0	>0.05
*Cry1*	Control	0.719	18.4	<0.001
	l-Orn	0.572	19.6	<0.001
*Cry2*	Control	0.182	11.6	>0.05
	l-Orn	0.425	12.0	>0.05
*Dec1*	Control	0.409	11.6	>0.05
	l-Orn	0.400	12.0	>0.05
*Dec2*	Control	0.692	6.8	<0.01
	l-Orn	1.322	8.4	<0.05
*Dbp*	Control	1.226	8.0	<0.05
	l-Orn	2.288	9.6	>0.05
*Rev-erbα*	Control	0.558	8.0	<0.001
	l-Orn	0.905	9.2	<0.01

**Table 2:** List of primers used in transcript analysis.

Target gene	Primer sequence (5′-3′)
*P0*-F	GCGTCCTGGCATTGTCTGTG
*P0*-R	TCCTCATCTGATTCCTCCGACTC
*Per2*-F	TGTGTGCTTACACGGGTGTCCTA
*Per2*-R	ACGTTTGGTTTGCGCATGAA
*Bmal1*-F	TGGGGCTGGACGAAGACAATGAGC
*Bmal1*-R	CGCCAAAATAGCTGTCGCCCTCTGA
*Cry1*-F	ATCCGCTGCGTCTATATCCTC
*Cry1*-R	CCCGAATCACAAACAGACG
*Cry2*-F	ACCCACGGCCCATCGTCAATCA
*Cry2*-R	GCTGATGCTCCCAGCTTGGCTTGA
*Clock*-F	AGCAGCAGCCACCACAGCAACA
*Clock*-R	TCGAAGGATTCCCGTGGAGCAACC
*Dbp*-F	TGCAGGGAAACAGCAAGCCCAAAGA
*Dbp*-R	CCACAGCAGCGGCGCAAAAAGA
*Per1*-F	TGCCTCGGGCCCTTGATGTGAT
*Per1*-R	CCAATTTGGGTCTGGGCCTCCTCA
*Dec1*-F	TTCGCCGTGGGAGAACGTGTCA
*Dec1*-R	CAGTGGCCGATGGTGGGATGAGAT
*Dec2*-F	CGCCCTTCTGCCTGCCCTTCTATCT
*Dec2*-R	GCACGGACGACAAGCAAGGGAAA
*Rev-erbα*-F	CGGATTCCCAGGAACATGGAGCA
*Rev-erbα*-R	GGCATGGCCGTTTGGGTAATGC
