# Supplementary material for: l-Ornithine affects peripheral clock gene expression in mice
Source: Sci Rep. 2016 Oct 5;6:34665. doi: 10.1038/srep34665 (PMC5050418; doi:10.1038/srep34665)
Supplement: Supplementary Information [file srep34665-s1.pdf]

Title

L-Ornithine affects peripheral clock gene expression in mice

Authors

Takafumi Fukuda[1,*], Atsushi Haraguchi[2], Mari Kuwahara[2], Kaai Nakamura[2], Yutaro Hamaguchi[2], Yuko Ikeda[2], Yuko Ishida[1], Guanying Wang[1], Chise Shirakawa[1], Yoko Tanihata[1], Kazuaki Ohara[1], and Shigenobu Shibata[2, *]

Addresses

[1] Research Laboratories for Health Science & Food Technologies, Kirin Company, Ltd., Yokohama, Kanagawa, Japan.

[2] Laboratory of Physiology and Pharmacology, School of Advanced Science and Engineering, Waseda University, Tokyo, Japan.

Supplementary Figure S1

Rhythm of clock gene mRNA expression in the sub gla detected by time series RT-PCR

(a) Experimental schedule (as in Fig. 2a).

(b) Clock gene mRNA levels in the sub gla determined by RT-PCR plotted against ZT. All values are expressed as means ± SEM (n=5-9 mice per group).

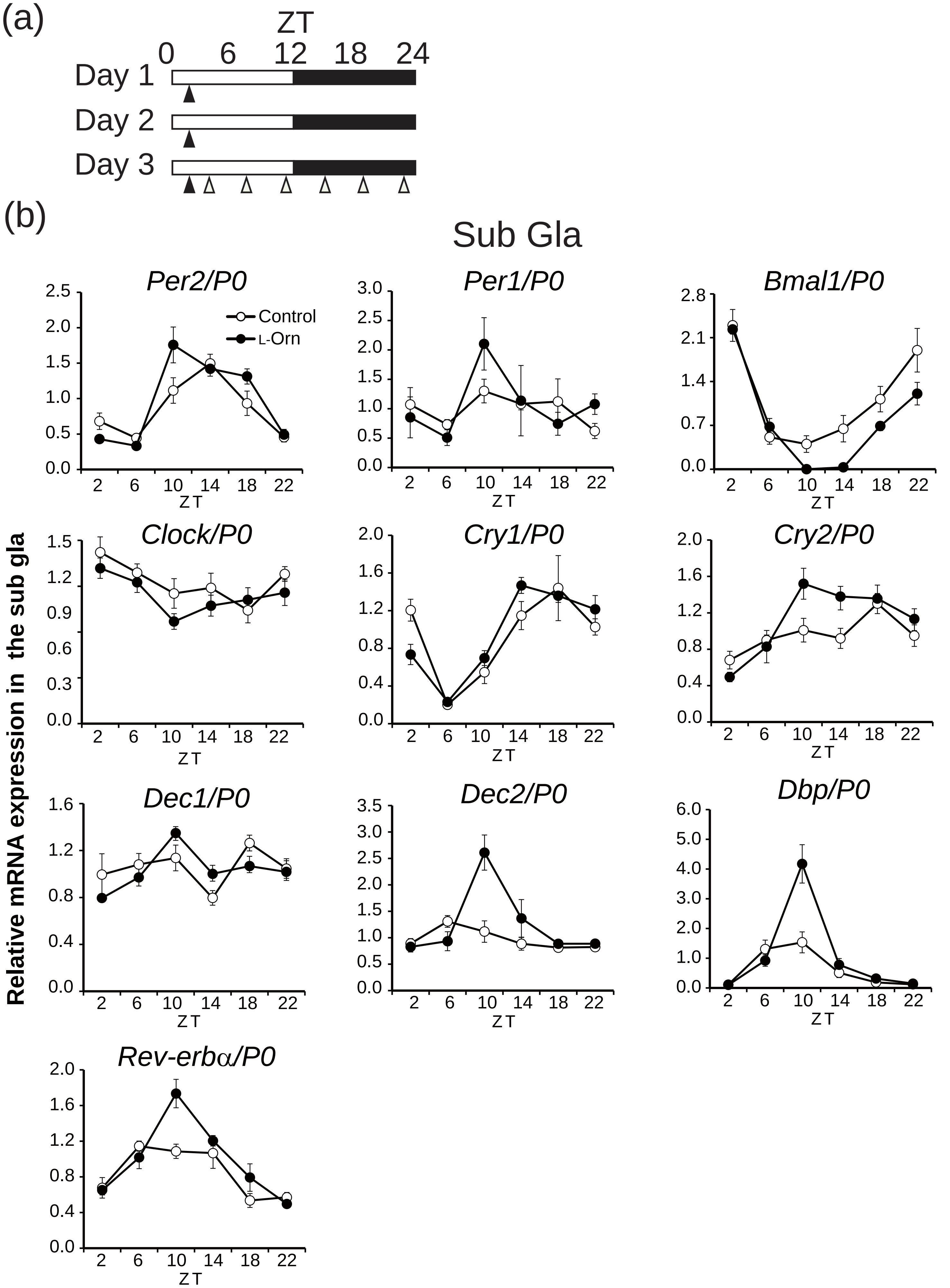

(a)
ZT
0 6 12 18 24
Day 1
Day 2
Day 3
(b)
Sub Gla
Per2/P0
Per1/P0
Bmal1/P0
Clock/P0
Cry1/P0
Cry2/P0
Dec1/P0
Dec2/P0
Dbp/P0
Rev-erbα/P0
Control
L-Orn
Relative mRNA expression in the sub gla
ZT

Supplementary Figure S2

Rhythm of clock gene mRNA expression in the kidney detected by time series RT-PCR

(a) Experimental schedule (as in Fig. 2a).

(b) Clock genes mRNA levels in the kidney determined by RT-PCR plotted against ZT. All values are expressed as means ± SEM (n=5-9 mice per group).

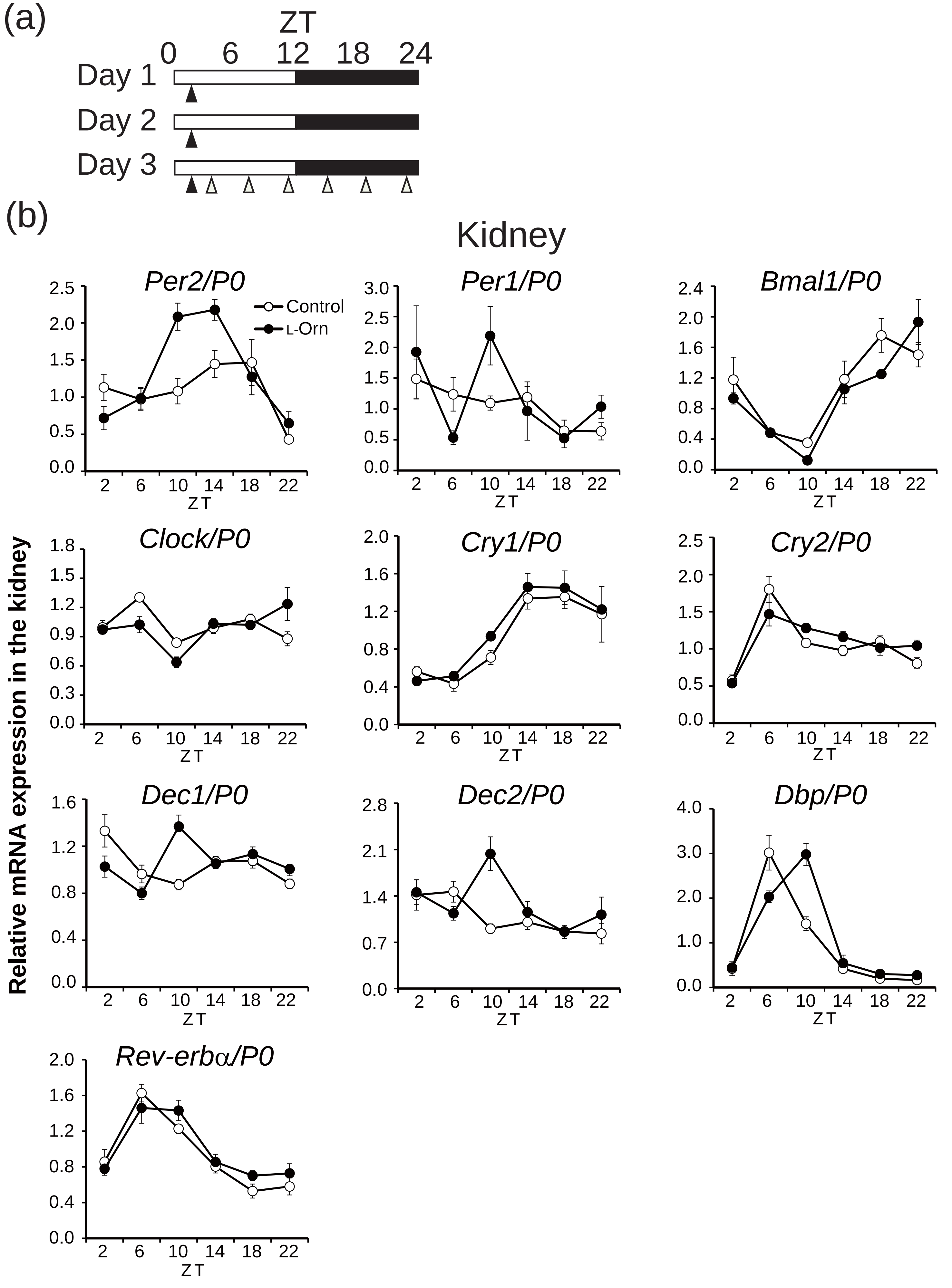

(a)
ZT
0 6 12 18 24
Day 1
Day 2
Day 3
(b)
Kidney
Relative mRNA expression in the kidney
Per2/P0
Control
L-Orn
Per1/P0
Bmal1/P0
Clock/P0
Cry1/P0
Cry2/P0
Dec1/P0
Dec2/P0
Dbp/P0
Rev-erbα/P0
ZT
2 6 10 14 18 22

Supplementary Figure S3

Effects of L-ornithine, L-glutamic acid and L-argine administration at ZT 1 on secretion of insulin and the expression level of m*Per2*

(a) Experimental schedule (as in Fig. 4a).

(b) RT-PCR analysis of clock genes in the liver (mean ± SEM, n=10 mice per group). mRNA expression was normalized to *P0*.

(c) Insulin concentration in plasma (mean ± SEM, n=10 mice per group). **P <0.01, *P<0.05 pairwise comparisons of all groups (Steel-Dwass test).

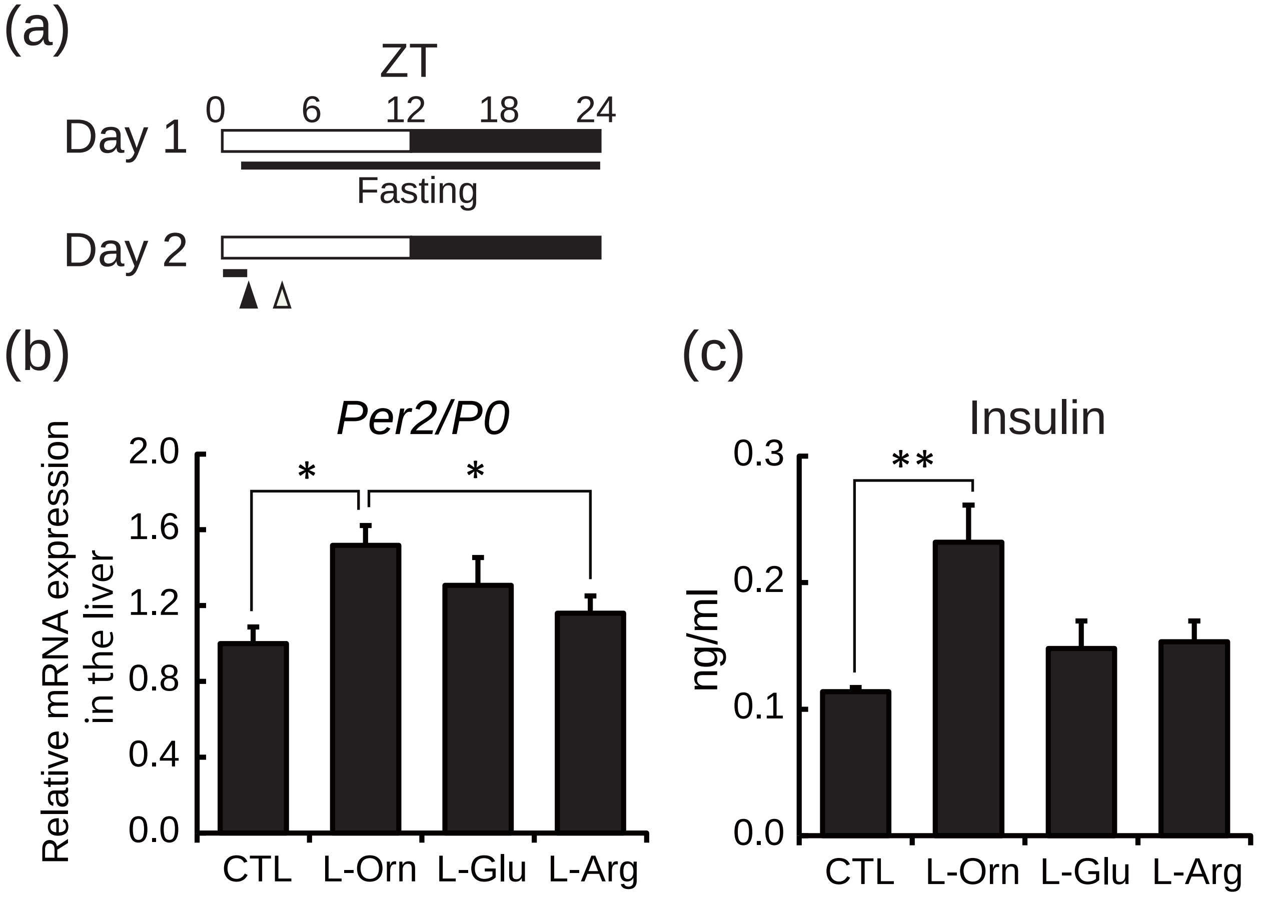
(a)
ZT
0
6
12
18
24
Day 1
Fasting
Day 2
(b)
Per2/P0
Relative mRNA expression in the liver
2.0
1.6
1.2
0.8
0.4
0.0
*
*
CTL
L-Orn
L-Glu
L-Arg
(c)
Insulin
ng/ml
0.3
0.2
0.1
0.0
**
CTL
L-Orn
L-Glu
L-Arg

Supplementary Table S1 List of amplitudes, acrophases and *P* values (representing precision of the fit) of the time series RT-PCR data in Supplementary Fig. S1 calculated by the cosinor procedure program

Sub Gla				
Clock Gene	Group	Amplitude	Acrophase	*P* value
Per2	Control	0.525	13.6	<0.05
	L-Orn	0.712	13.6	<0.02
Per1	Control	0.340	12.4	>0.05
	L-Orn	0.798	11.6	>0.05
Bmal1	Control	0.947	22.0	<0.05
	L-Orn	1.116	2.0	<0.05
Clock	Control	0.190	2.8	>0.05
	L-Orn	0.174	2.0	>0.05
Cry1	Control	0.619	19.2	<0.05
	L-Orn	0.618	17.6	<0.005
Cry2	Control	0.311	16.0	>0.05
	L-Orn	0.513	14.4	<0.02
Dec1	Control	0.234	22.0	>0.05
	L-Orn	0.277	12.4	>0.05
Dec2	Control	0.247	9.2	<0.02
	L-Orn	0.891	10.8	>0.05
Dbp	Control	0.710	8.8	<0.02
	L-Orn	2.037	10.0	>0.05
Rev-erbα	Control	0.305	9.2	<0.01
	L-Orn	0.619	10.8	<0.005

Supplementary Table S2 List of amplitudes, acrophases and *P* values (representing precision of the fit) of the time series RT-PCR data in Supplementary Fig. S2 calculated by the cosinor procedure program

Kidney				
Clock Gene	Group	Amplitude	Acrophase	*P* value
Per2	Control	0.519	13.6	>0.05
	L-Orn	0.768	12.4	<0.001
Per1	Control	0.425	6.4	>0.05
	L-Orn	0.832	6.4	>0.05
Bmal1	Control	0.702	20.0	<0.001
	L-Orn	0.905	20.8	<0.01
Clock	Control	0.233	5.2	>0.05
	L-Orn	0.299	21.6	>0.05
Cry1	Control	0.460	17.2	<0.001
	L-Orn	1.007	16.4	<0.001
Cry2	Control	0.614	8.8	>0.05
	L-Orn	0.465	10.8	>0.05
Dec1	Control	0.228	2.0	>0.05
	L-Orn	0.284	13.6	>0.05
Dec2	Control	0.317	4.8	>0.05
	L-Orn	0.59	8.0	>0.05
Dbp	Control	1.426	7.2	>0.05
	L-Orn	1.352	8.4	<0.05
Rev-erbα	Control	0.549	7.2	<0.005
	L-Orn	0.379	8.0	<0.02
